# Spacer effect on nanostructures and self-assembly in organogels via some bolaform cholesteryl imide derivatives with different spacers

**DOI:** 10.1186/1556-276X-8-406

**Published:** 2013-10-02

**Authors:** Tifeng Jiao, Fengqing Gao, Qingrui Zhang, Jingxin Zhou, Faming Gao

**Affiliations:** 1 Hebei Key Laboratory of Applied Chemistry, School of Environmental and Chemical Engineering, Yanshan University, Qinhuangdao 066004, China; 2 State Key Laboratory of Solid Lubrication, Lanzhou Institute of Chemical Physics, Chinese Academy of Sciences, Lanzhou 730000, China

**Keywords:** Organogel, Nanostructures, Self-assembly, Spacer effect, Imide derivative, Cholesteryl

## Abstract

In this paper, new bolaform cholesteryl imide derivatives with different spacers were designed and synthesized. Their gelation behaviors in 23 solvents were investigated, and some of them were found to be low molecular mass organic gelators. The experimental results indicated that these as-formed organogels can be regulated by changing the flexible/rigid segments in spacers and organic solvents. Suitable combination of flexible/rigid segments in molecular spacers in the present cholesteryl gelators is favorable for the gelation of organic solvents. Scanning electron microscopy and atomic force microscopy observations revealed that the gelator molecules self-assemble into different aggregates, from wrinkle and belt to fiber with the change of spacers and solvents. Spectral studies indicated that there existed different H-bond formations between imide groups and assembly modes, depending on the substituent spacers in molecular skeletons. The present work may give some insight into the design and character of new organogelators and soft materials with special molecular structures.

## Background

Organogels, which are various three-dimensional (3D) aggregates with micrometer-scale lengths and nanometer-scale diameters immobilizing the flow of liquids, have been well known for wide applications on materials, drug delivery, agents, and sensors as well as water purification in recent years [1-8]. The driving forces responsible for gel formations are specific or non-covalent interactions such as the dipole-dipole interaction, van der Waals forces, hydrogen bonding, π-π stacking, and host-guest interaction [9-14]. In particular, complementary hydrogen bonding patterns play a very important role in forming various architectures, and their application in the fabrication of organogels has been attempted [15-17]. In addition, although gels are early found in polymer systems, there has recently been an increasing interest in low molecular mass organic gelators (LMOGs) [18-20]. Such organogels have some advantages over polymer gels: the molecular structure of the gelator is defined, and the gel process is usually reversible. Such properties make it possible to design various functional gel systems and produce more complicated and controllable nanostructures [21-25].

Recently, cholesterol-based imide derivatives have been reported as a new class of organogelator architectures because of their unique directional self-association through van der Waals interactions in the aggregates of the gelators [26]. For example, Shinkai and co-workers prepared a number of dicholesterol derivatives bearing various functional linkers as versatile gelators [27-32] and obtained inorganic materials possessing unique structures by using the corresponding gels as templates. In our reported work, the gelation properties of some cholesterol imide derivatives consisting of cholesteryl units and photoresponsive azobenzene substituent groups have been investigated [33]. We found that a subtle change in the headgroup of azobenzene segment can produce a dramatic change in the gelation behavior of both compounds. In addition, the gelation properties of bolaform and trigonal cholesteryl derivatives with different molecular skeletons have been characterized [34]. Therein, we have investigated the effect of molecular shapes on the microstructures of such organogels and found that various kinds of hydrogen bond interactions among the molecules play an important role in the formation of gels.

As a continuous work, herein, we have designed and synthesized some bolaform cholesteryl imide derivatives with different spacers. In all compounds, the diphenyl group, alkyl chains, or hydrophilic imine groups in spacers linked by ether band were symmetrically attached to cholesterol substituent headgroups to show bolaform molecular skeletons. We have found that most of the compounds could form different organogels in various organic solvents. Characterization of these organogels by scanning electron microscopy (SEM) and atomic force microscopy (AFM) revealed different structures of the aggregates in the gels. We have investigated the effect of spacers in gelators on the microstructures of such organogels in detail and found different kinds of hydrogen bond interactions between imide groups and assembly units.

## Methods

### Materials

**Figure 1:**
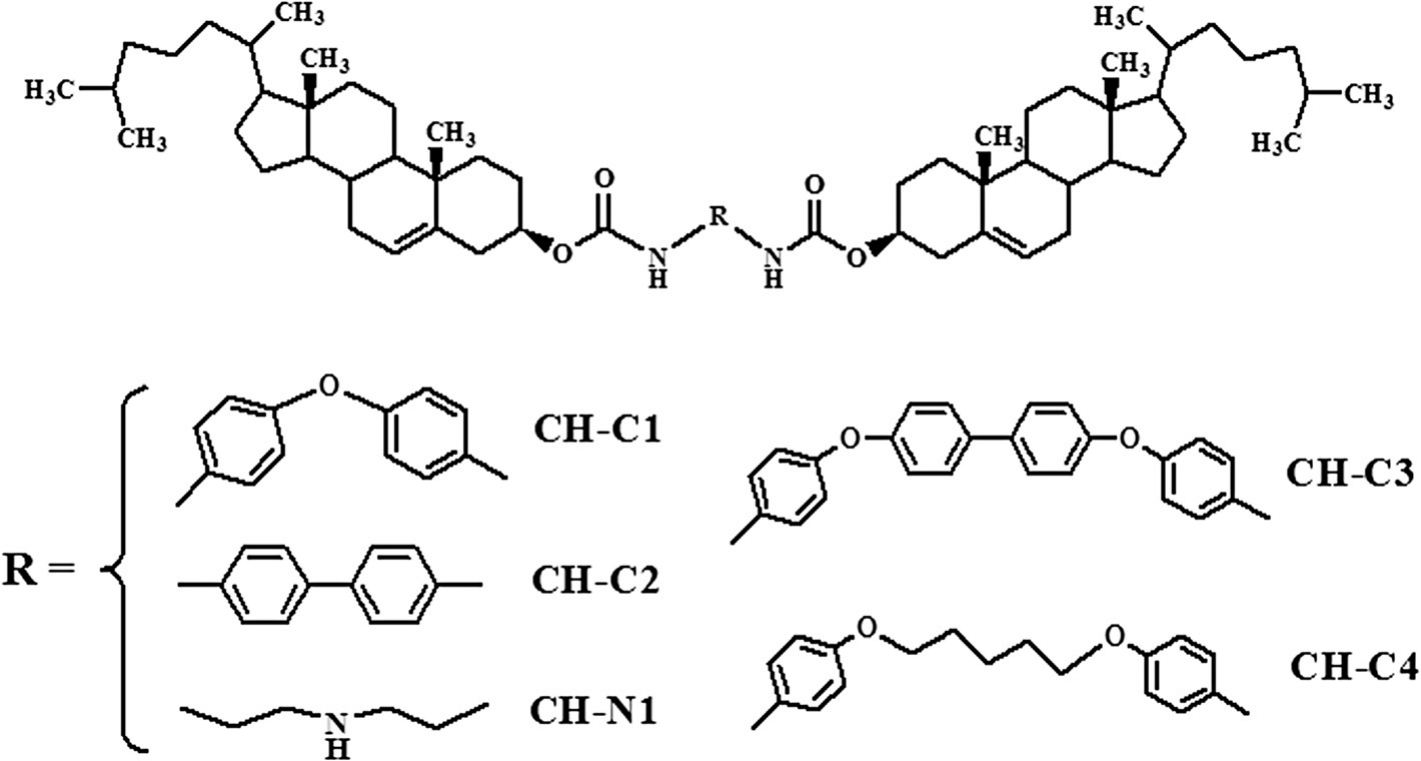
Structures and abbreviations of bolaform cholesteryl imide derivatives with different spacers.

The starting materials, cholesteryl chloroformate, benzidine, diethylenetriamine, 1,5-bis(4-aminophenoxy)pentane, 4,4′-diaminodiphenyl ether, and 4,4′-(1,1′-biphenyl-4,4′-diyldioxy)dianiline, were purchased from TCI Chemicals (Shanghai, China), Alfa Aesar (Beijing, China), or Sigma-Aldrich Chemicals (Shanghai, China). Other used reagents shown in Table 1 were all of analysis purity from Beijing Chemicals and were distilled before use. Deionized water was used in all cases. Then, these cholesteryl imide derivatives were synthesized by a similar method according to our previous report [34]. The products were purified by recrystallization in an ethanol solution as pale solids. The final products and their abbreviations are shown in Figure 1, which were confirmed by ^1^H NMR and elemental analysis.

**Table 1:** Gelation behaviors of the cholesteryl derivatives at room temperature

**Solvents**	**CH-C1**	**CH-C2**	**CH-C3**	**CH-C4**	**CH-N1**
*n*-Propanol	PS	PS	PS	PS	S
Isopropanol	S	PS	PS	PS	S
*n*-Butanol	PS	S	PS	PS	S
*n*-Pentanol	PS	PS	PS	PS	S
Isopentanol	PS	PS	PS	PS	PS
Isooctanol	G (1.5)	S	PS	PS	S
Acetone	PS	PS	PS	S	PS
Cyclopentanone	S	PS	PS	PS	S
Cyclohexanone	S	PS	G (2.0)	S	S
*n*-Hexane	G (1.5)	PS	PS	PS	S
1,4-Dioxane	G (1.5)	PS	G (2.0)	S	S
Benzene	S	PS	PS	S	PS
Toluene	S	PS	PS	S	S
Nitrobenzene	G (1.5)	PS	G (1.5)	G (1.5)	S
Aniline	G (1.5)	PS	PS	G (2.0)	S
Ethanolamine	I	I	I	I	S
Ethyl acetate	PS	PS	G (2.0)	S	S
*n*-Butyl acrylate	PS	PS	PS	G (2.0)	S
Acetonitrile	PS	PS	S	S	S
THF	S	S	S	S	S
Pyridine	S	PS	S	S	G (2.5)
Petroleum ether	PS	PS	G (2.0)	S	PS
DMF	PS	PS	G (1.5)	G (1.5)	S

DMF, dimethylformamide; THF, tetrahydrofuran; S, solution; PS, partially soluble; G, gel; I, insoluble. For gels, the critical gelation concentrations at room temperature are shown in parentheses, (*w*/*v*)%.

### Gel preparation

At present, five kinds of cholesteryl imide derivatives with different spacers were tested to prepare possible organogels according to a simple procedure. Firstly, a weighted amount of imide compounds and a measured volume of selected pure organic solvent were placed into a sealed glass bottle, and the solution was ultrasonicated in a sonic bath for 15 min in order to obtain good dispersion. After that, the solution was heated in a water bath at a temperature of 80°C for 15 min. Then, the solution was cooled to room temperature in air, and the test bottle was inversed to see if a gel was formed. At this stage, G, S, PS, and I were denoted to character the states of imide derivatives, indicating gel, solution, a few precipitates, and insoluble systems, respectively. Critical gelation concentration refers to the minimum concentration of the gelator for gel formation.

### Characterization

These prepared organogels under the critical gelation concentration were dried using a vacuum pump for more than 12 h to remove solvents and form xerogels. Then, the obtained xerogel samples were attached to different substrates, such as mica, copper foil, glass, and CaF_2_ slice for morphological and spectral investigation. AFM data were measured using a Nanoscope VIII Multimode Scanning Probe Microscope (Veeco Instrument, Plainview, NY, USA) with silicon cantilever probes. All AFM images were shown in the height mode without any image processing except flattening. SEM images of the xerogels were measured on a Hitachi S-4800 field emission scanning electron microscope with an accelerating voltage of 5 to 15 kV. For SEM measurement, the samples were coated on copper foil fixed by conductive adhesive tape and shielded by gold nanoparticles. The X-ray diffraction (XRD) pattern was measured using a Rigaku D/max 2550PC diffractometer (Rigaku Inc., Tokyo, Japan) with a CuKα radiation wavelength of 0.1542 nm under a voltage of 40 kV and a current of 200 mA. Fourier transform infrared (FT-IR) spectra were obtained using a Nicolet is/10 FT-IR spectrophotometer from Thermo Fisher Scientific Inc. (Waltham, MA, USA) by average 32 scans and at a resolution of 4 cm^-1^.

## Results and discussion

**Figure 2:**
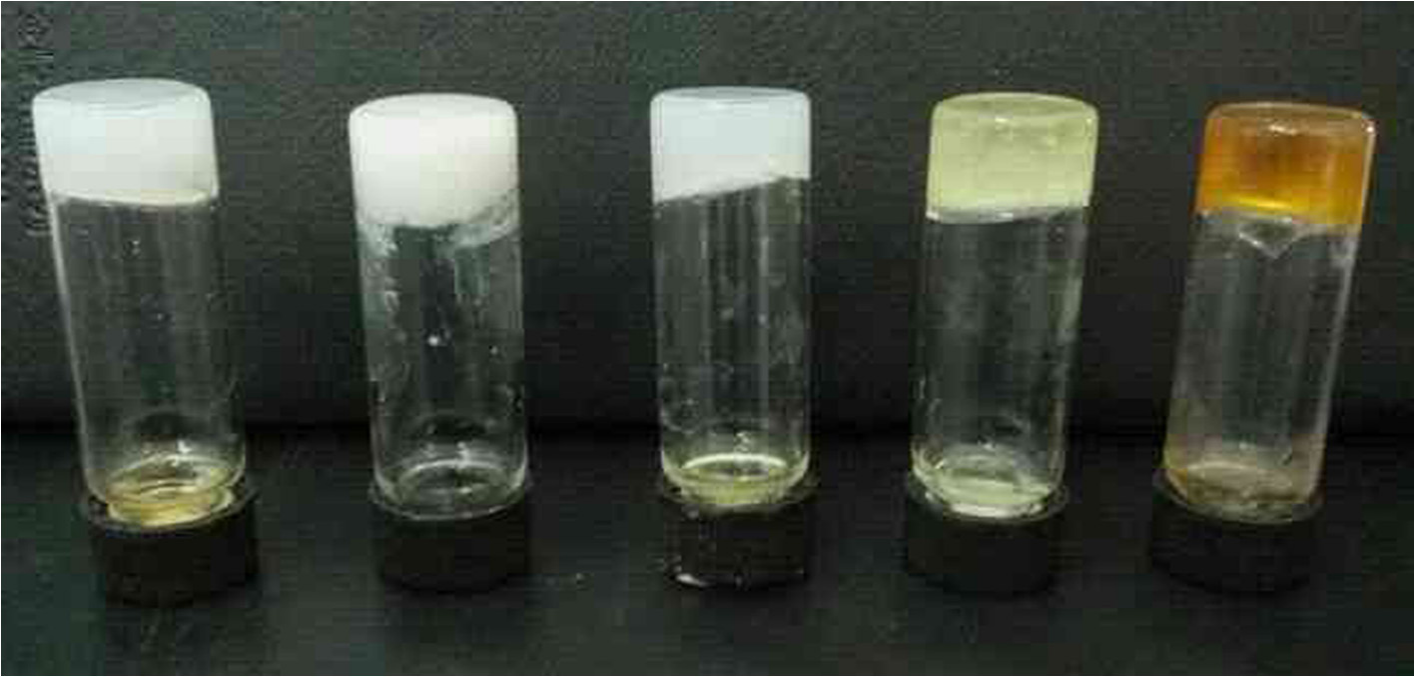
**Photographs of CH-C1 organogels in different solvents: isooctanol, *n-*hexane, 1,4-dioxane, nitrobenzene, and aniline (from left to right).**

The gelation performances of all compounds in 23 solvents are listed in Table 1. Examination of the table reveals that all compounds are efficient gelators except CH-C2. Firstly, CH-C1 can gel in five kinds of solvents, such as isooctanol, *n*-hexane, 1,4-dioxane, nitrobenzene, and aniline. The corresponding photographs of organogels of CH-C1 in different solvents are shown in Figure 2. As for CH-C3 with an additional diphenyl group linked by ether band in the spacer part, six kinds of organogels were formed. In addition, as for CH-C4 with a five-carbon alkyl substituent chain linked by phenoxy ether band in the molecular spacer, the number of formed organogels shifted to 4. Furthermore, for the case of CH-N1 with a hydrophilic diethylene spacer containing an amino group, only one kind of organogel can form in pyridine. The present data shown in Table 1 indicate that change of spacer groups in molecular skeletons can have a profound effect on the gelation abilities of the studied imide compounds, which is similar to some systems in our previous reports about organogels [24,34-36]. It seemed that the suitable combination of flexible/rigid segments in molecular spacers in the present cholesteryl gelators is favorable for the gelation of organic solvents. In addition, the stereo effect of phenoxy groups on intermolecular π-π stacking in the gel formation process is also obvious for all cases except CH-N1. Moreover, it should be noted that for some of the present gelators, CH-C1, CH-C3, and CH-C4 can form organogels in nitrobenzene. The change of gelation behaviors can be attributed to the change of the spatial conformation and intermolecular forces of the gelators due to different spacers in molecular skeletons, which may increase the ability of the gelator molecules to self-assemble into ordered structures, a necessity for forming organized three-dimensional network structures.

**Figure 3:**
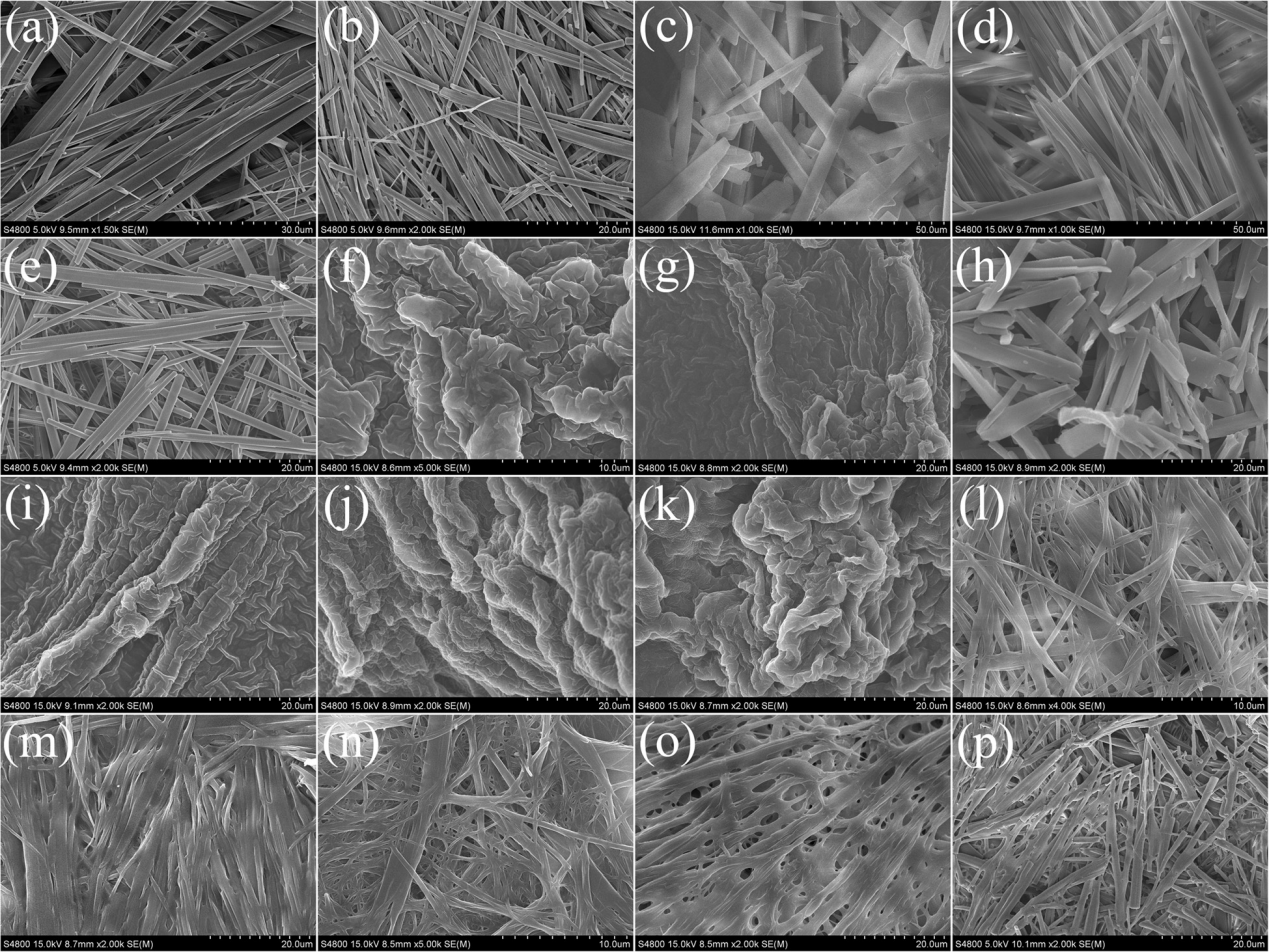
**SEM images of xerogels.** CH-C1 gels (**(a)** isooctanol, **(b)***n*-hexane, **(c)** 1,4-dioxane, **(d)** nitrobenzene, **(e)** aniline), CH-C3 gels (**(f)** cyclohexanone, **(g)** 1,4-dioxane, **(h)** nitrobenzene, **(i)** ethyl acetate, **(j)** petroleum ether, **(k)** DMF), CH-C4 gels (**(l)** nitrobenzene, **(m)** aniline, **(n)***n*-butyl acrylate, **(o)** DMF), and CH-N1 gels (**(p)** pyridine).

**Figure 4:**
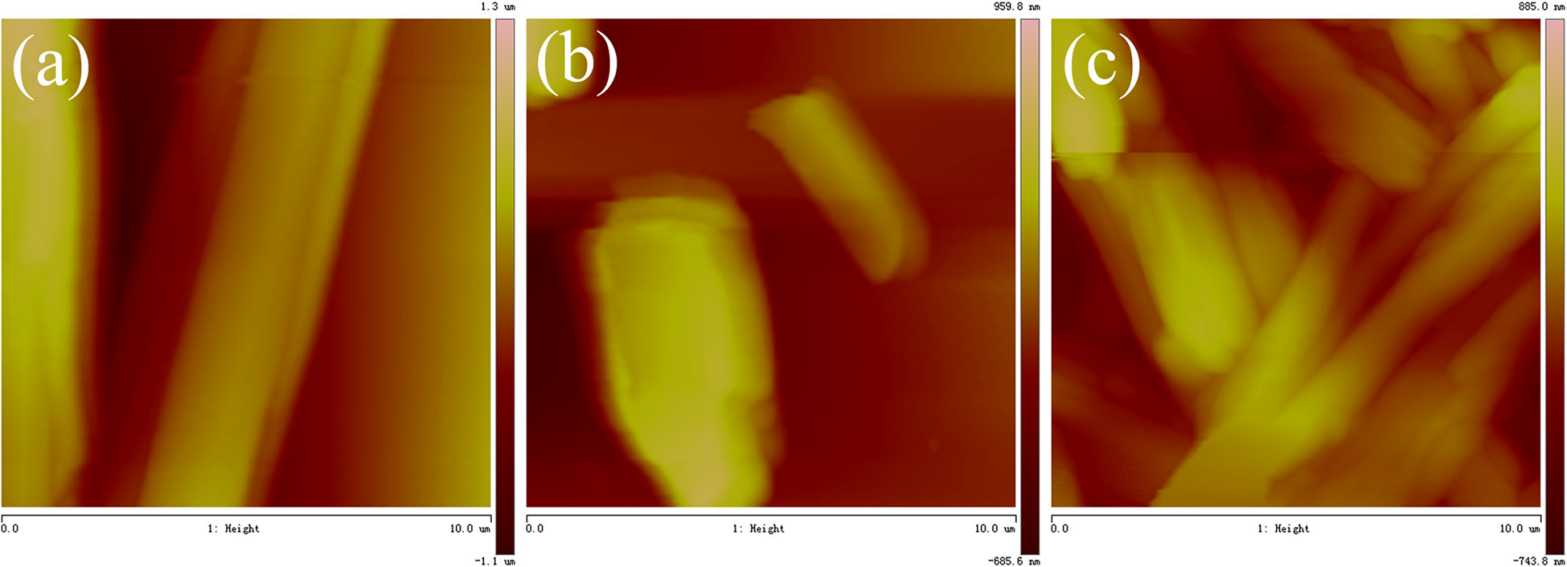
**AFM images of xerogels. (a)** CH-C1, **(b)** CH-C3, and **(c)** CH-C4 gels in nitrobenzene.

Many researchers have reported that a gelator molecule constructs nanoscale superstructures such as nanofibers, nanoribbons, and nanosheets in a supramolecular gel [37-39]. To obtain a visual insight into the present gel microstructures, the typical nanostructures of these gels were studied by SEM and AFM techniques, as shown in Figures 3 and 4. From the present diverse images, it can be easily investigated that the microstructures of the xerogels of all mixtures in different solvents are significantly different from each other, and the morphologies of the aggregates change from wrinkle and belt to fiber with change of solvents and gelators. Besides, more wrinkle-like aggregates with different sizes were prepared in gels of CH-C3 with an additional diphenyl group linked by ether band in the spacer part. Furthermore, the xerogels of CH-C1, CH-C3, and CH-C4 in nitrobenzene were characterized by AFM, as shown in Figure 4. From the images, it is interesting to note that morphologies of fiber, rod, and belt with different sizes were observed for the three xerogels, respectively. The morphologies of the aggregates shown in the SEM and AFM images may be rationalized by considering a commonly accepted idea that highly directional intermolecular interactions, such as hydrogen bonding or π-π interactions, favor formation of organized belt or fiber micro/nanostructures [40-42]. The differences of morphologies between different gelators can be mainly due to the different strengths of the hydrophobic force between cholesteryl segments, π-π stacking, and stereo hindrance between flexible/rigid segments in molecular spacers, which have played an important role in regulating the intermolecular orderly stacking and formation of special aggregates.

**Figure 5:**
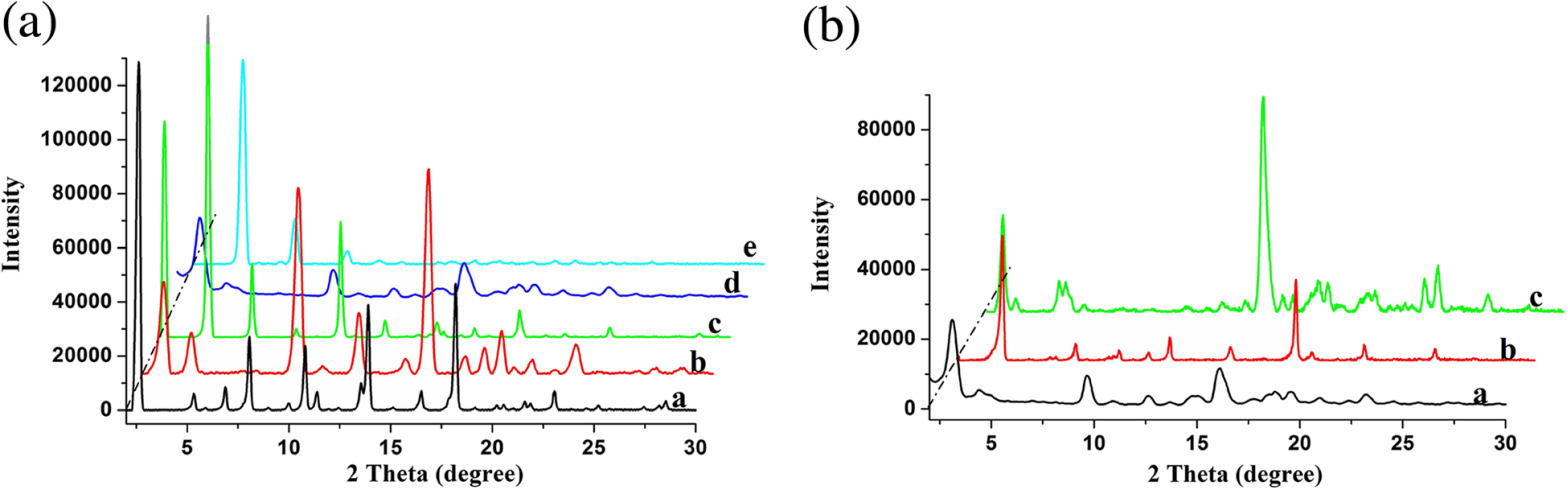
**X**-**ray diffraction patterns of xerogels. (a)** CH-C1 (a, isooctanol; b, *n*-hexane; c, 1,4-dioxane; d, nitrobenzene; and e, aniline); **(b)** a, CH-C1; b, CH-C3; and c, CH-C4, in nitrobenzene.

In addition, with the purpose of investigating the orderly stacking of xerogel nanostructures, XRD patterns of all xerogels from gels were measured. Firstly, the data of CH-C1 were taken as an example, as shown in Figure 5a. The curve of CH-C1 xerogel from 1,4-dioxane shows main peaks in the angle region (2*θ* values, 2.17°, 4.32°, 6.53°, and 10.84°) corresponding to *d* values of 4.07, 2.04, 1.35, and 0.82 nm, respectively. The corresponding *d* values follow a ratio of 1:1/2:1/3:1/5, suggesting a lamellar-like structure of the aggregates in the gel [43]. As for the curves of CH-C1 in other solvents, isooctanol, *n*-hexane, nitrobenzene, and aniline, the minimum 2*θ* values are 2.62°, 3.02°, 3.08°, and 4.36°, corresponding to *d* values of 3.37, 2.93, 2.87, and 2.03 nm, respectively. The change of values can be mainly attributed to the different assembly modes of the gelator in various solvents. Furthermore, the curves of CH-C1, CH-C3, and CH-C4 in nitrobenzene were also compared to investigate the spacer effects on assembly modes. Minimum 2*θ* peaks were observed at 4.14° and 2.74° for CH-C3 and CH-C4, respectively. The corresponding *d* values are 2.14 and 3.23 nm, respectively. The XRD results demonstrated again that the spacers had great effects on the assembly modes of these imide gelators.

**Figure 6:**
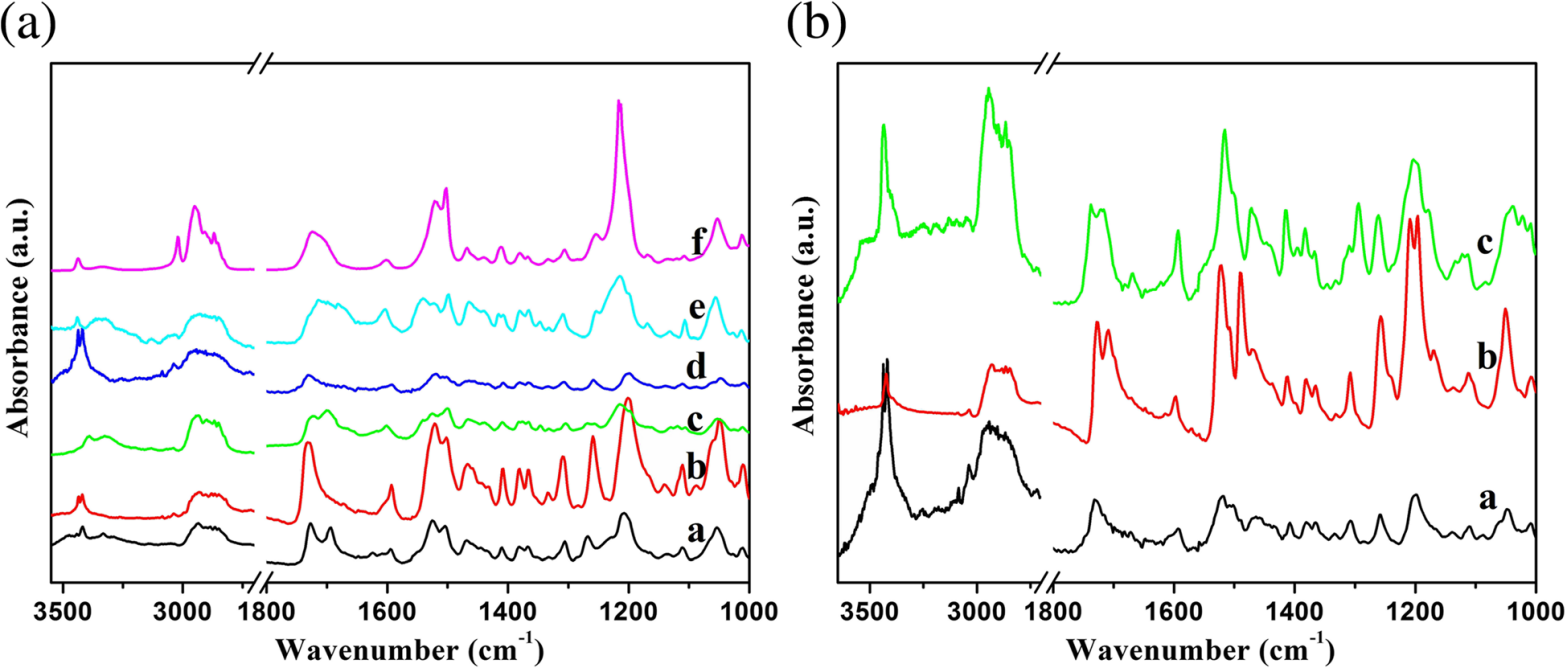
**FT-IR spectra of xerogels. (a)** CH-C1 (a, isooctanol; b, *n*-hexane; c, 1,4-dioxane; d, nitrobenzene; e, aniline; and f, chloroform solution); **(b)** a, CH-C1; b, CH-C3; and c, CH-C4, in nitrobenzene.

It is well known that hydrogen bonding plays an important role in the self-assembly process of organogels [44,45]. At present, we have measured the FT-IR spectra of xerogels of all compounds in order to further and investigate the assembly process. Firstly, the xerogels of CH-C1 were taken as examples, as shown in Figure 6a. As far as the spectrum of CH-C1 xerogel in nitrobenzene, some main peaks were observed at 3,436, 3,415, 1,728, and 1,593 cm^-1^. These bands can be attributed to the N-H stretching, C=O stretching of ester, amide I band, and benzene ring, respectively [34,46,47]. These bands indicate H-bond formation between intermolecular amide and carbonyl groups in the gel state. The spectra of other xerogels in different solvents are different, suggesting the different H-bond and assembly modes of the gelator in various solvents. In addition, it is interesting to note that the spectra of xerogels of CH-C1, CH-C3, and CH-C4 in nitrobenzene were compared in Figure 6b, showing an obvious change. The main peaks attributed to the C=O stretching of ester and the amide I band shifted to 1,726 and 1,707 as well as 1,735 and 1,716 cm^-1^ for CH-C3 and CH-C4, respectively. This implied that there were differences in the strength and direction of the intermolecular hydrogen-bond interactions in these xerogels. The present data further verified that the spacer in molecular skeletons can regulate the stacking of the gelator molecules to self-assemble into ordered structures by distinct intermolecular hydrogen bonding.

**Figure 7:**
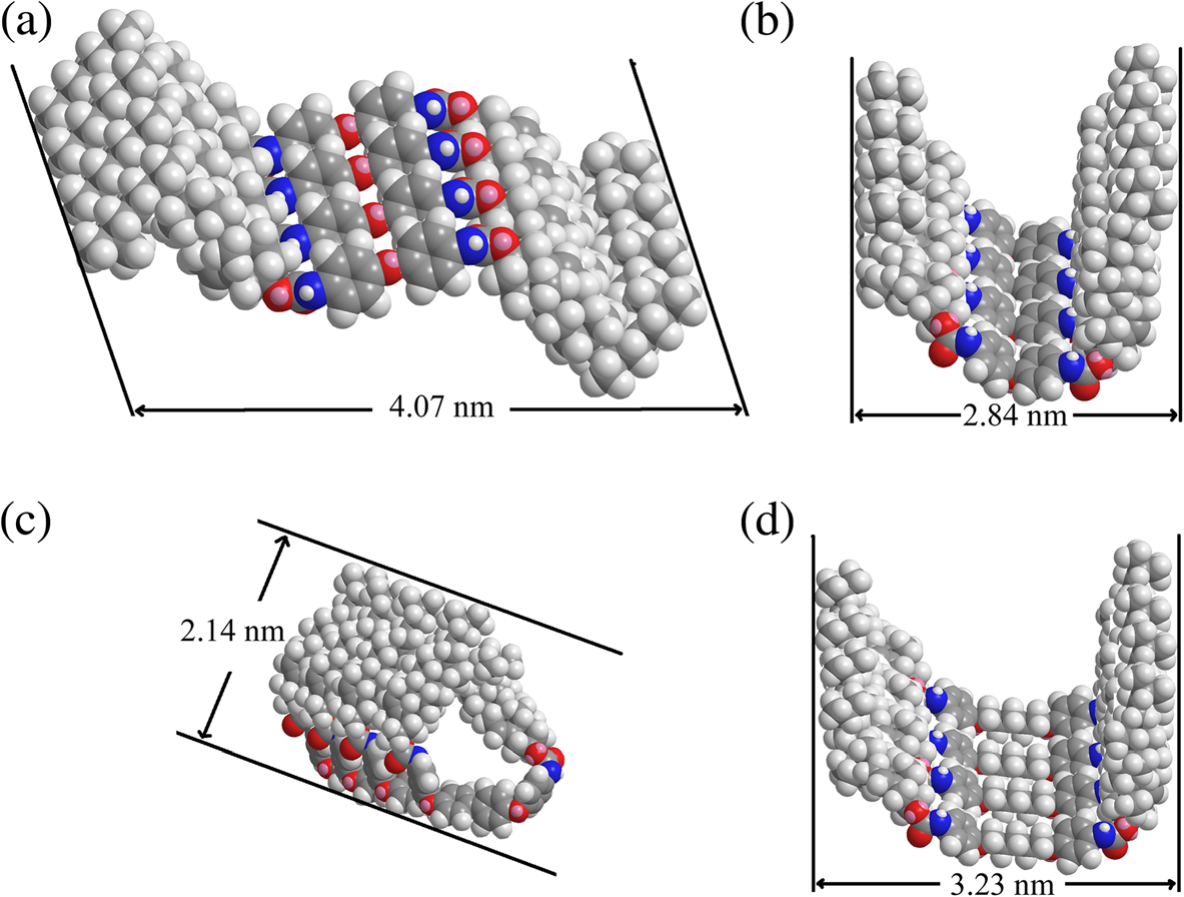
**Rational assembly modes of CH-C1, CH-C3, and CH-C4 in gels.** Experimental values of **(a, b)** CH-C1 in 1,4-dioxane and nitrobenzene, **(c)** CH-C3 in nitrobenzene, and **(d)** CH-C4 in nitrobenzene.

Considering the XRD results described above and the hydrogen bonding nature of the organized packing of these organogels as confirmed by FT-IR measurements, some possible packing modes of these gelators were proposed and schematically shown in Figure 7. As for CH-C1 xerogel from 1,4-dioxane, due to the flexibility of ether band in the molecular skeleton and different intermolecular forces with solvents, after the intermolecular hydrogen bonding and orderly stacking in different solvents, various repeating units with different lengths were obtained. So corresponding *d* values of 4.07 and 2.84 nm were obtained from 1,4-dioxane and nitrobenzene, respectively, as shown in Figure 7a,b. As for CH-C3 with an additional diphenyl group linked by ether band in the spacer part, the combination of a flexible ether band and a rigid diphenyl segment in the molecular spacer with π-π stacking seemed more suitable to adjust molecular conformation to self-assemble and form organized stacking nanostructures. The obtained experimental value of CH-C3 in nitrobenzene was 2.14 nm, which was near half of the calculated molecular length, suggesting a symmetrical stacking mode, shown in Figure 7c. In addition, for the case of CH-C4 with a five-carbon alkyl substituent chain linked by phenoxy ether band in the molecular spacer, due to the addition of a flexible alkyl segment and a weak hydrophobic force between alkyl chains, it can also stack and form some belt-like aggregates with a stacking length of 3.23 nm in nitrobenzene, as shown in Figure 7d. Moreover, for CH-C2 and CH-N1, the inefficient or poor gelation behaviors in the present solvents may be mainly attributed to the too rigid or too flexible spacers in molecular skeletons, which cannot cause enough intermolecular forces to make the molecules align and stack in an organized way to form various nanostructures. Meanwhile, it should be noted that this phenomenon can be compared with the results of our recent works [24,25,48]. Therein, functionalized imide derivatives with the substituent groups of cholesteryl, azobenzene, luminol, and benzimidazole/benzothiazole residue can have a profound effect on the gelation abilities and the as-formed nanostructures of the studied compounds. For the present gelators, the experimental data showed that the spacers in the molecular skeleton have played a crucial role in the gelation behavior of all gelators in various organic solvents. Suitable combination of flexible/rigid segments in molecular spacers in the present cholesteryl gelators is favorable for the gelation of organic solvents. Now, the drug release behaviors generated by the present xerogels in the mixture of Congo red are under investigation to display the relationship between the molecular structures of as-formed nanostructures and their properties.

## Conclusions

Five bolaform cholesteryl imide derivatives with different spacers have been synthesized. Their gelation behaviors in 23 kinds of organic solvents have been investigated. The formed organogels can be regulated by changing the flexible/rigid segments in spacers and organic solvents. Suitable combination of flexible/rigid segments in molecular spacers in the present cholesteryl gelators is favorable for the gelation of organic solvents. Morphological studies revealed that the gelator molecules self-assemble into different aggregates, from wrinkle and belt to fiber with the change of spacers and solvents. Spectral studies indicated that there existed different H-bond formations between imide groups and assembly modes, depending on the substituent spacers in molecular skeletons. The prepared nanostructures have wide perspectives and many potential applications in nanoscience and material fields due to their scientific values. These results afford useful information for the design and development of new versatile low molecular mass organogelators and soft matter.

## Competing interests

The authors declare that they have no competing interests.

## Authors’ contributions

TJ participated in the analysis and the testing of the nanostructures. FeG carried out the synthesis of compounds and characterization of organogels. QZ and FaG supervised this work, helped in the analysis and interpretation of data, and, together with JZ, worked on the drafting and revisions of the manuscript. TJ and QZ conceived of the study and participated in its design and characterization. JZ participated in the design of the study and provided analysis instruments. All authors read and approved the final manuscript.

## Authors’ information

TJ and QZ are associate professors. FeG is an MD student. FaG is a professor and the Dean of the School of Environmental and Chemical Engineering. JZ is a laboratory assistant in Yanshan University.
